# Quantitative Retrieval of Soil Salinity in Arid Regions: A Radar Feature Space Approach with Fully Polarimetric SAR Data

**DOI:** 10.3390/s25082512

**Published:** 2025-04-16

**Authors:** Ilyas Nurmemet, Aihepa Aihaiti, Yilizhati Aili, Xiaobo Lv, Shiqin Li, Yu Qin

**Affiliations:** 1 College of Geography and Remote Sensing Sciences, Xinjiang University, Urumqi 830046, China; 107552301167@stu.xju.edu.cn (A.A.); 107552201173@stu.xju.edu.cn (Y.A.); 107552201156@stu.xju.edu.cn (X.L.); 107552203693@stu.xju.edu.cn (S.L.); 107552301195@stu.xju.edu.cn (Y.Q.); 2 Xinjiang Key Laboratory of Oasis Ecology, Xinjiang University, Urumqi 830046, China; 3 Xinjiang Field Scientific Observation and Research Station for the Oasisization Process in the Hinterland of the Taklamakan Desert, Yutian 848400, China

**Keywords:** soil salinity, feature space, polarimetric decomposition, Gaofen-3, synthetic aperture radar, arid regions

## Abstract

Soil salinization is a critical factor affecting land desertification and limiting agricultural development in arid regions, and the rapid acquisition of salinized soil information is crucial for prevention and mitigation efforts. In this study, we selected the Yutian Oasis in Xinjiang, China as the study area and utilized Gaofen-3 synthetic aperture radar (SAR) remote sensing data and field measurements to analyze the correlations between the salinized soil properties and 36 polarimetric radar feature components. Based on the analysis results, two components with the highest correlation, namely, Yamaguchi4_vol (*p* < 0.01) and Freeman3_vol (*p* < 0.01), were selected to construct a two-dimensional feature space, named Yamaguchi4_vol-Freeman3_vol. Based on this feature space, a radar salinization monitoring index (RSMI) model was developed. The results indicate that the RSMI exhibited a strong correlation with the surface soil salinity, with a correlation coefficient of 0.85. The simulated values obtained using the RSMI model were well-fitted to the measured soil electrical conductivity (EC) values, achieving an R^2^ value of 0.72 and a root mean square error (RMSE) of 7.28 dS/m. To assess the model’s generalizability, we applied the RSMI to RADARSAT-2 SAR data from the environmentally similar Weiku Oasis. The validation results showed comparable accuracy (R^2^ = 0.70, RMSE = 9.29 dS/m), demonstrating the model’s robustness for soil salinity retrieval across different arid regions. This model offers a rapid and reliable approach for quantitative monitoring and assessment of soil salinization in arid regions using fully polarimetric radar remote sensing. Furthermore, it lays the groundwork for further exploring the application potential of Gaofen-3 satellite data and expanding its utility in soil salinization monitoring.

## 1. Introduction

Soil salinization is a critical global environmental issue that significantly threatens soil health, agricultural productivity, and ecological sustainability, especially in arid and semi-arid regions [1]. It is estimated that nearly 20% of the world’s irrigated soils are affected by salinization, with this figure rising to 30% in arid and semi-arid areas [2,3]. In Xinjiang, China, serves as a crucial agricultural region, with saline-alkali soils accounting for approximately one-third of its arable land, a proportion that is far above the national average [4]. This extensive soil salinization not only reduces agricultural yield but also exacerbates land degradation, threatening food security and rural livelihoods [5]. Therefore, effective and timely monitoring of soil salinization is essential for developing sustainable management strategies aimed at mitigating its adverse effects and improving soil quality. Monitoring systems that provide accurate, large-scale, and real-time salinity data are crucial for the early detection of soil degradation, enabling better decision-making for soil reclamation and agricultural planning.

Traditional methods for monitoring soil salinity, such as soil sampling and laboratory analysis, electromagnetic induction (EMI) measurements, direct current (DC) resistivity, and ground penetrating radar (GPR) [6,7,8,9], provide reliable and accurate measurements of soil salinity, but they are time-consuming and labor-intensive. These methods generally provide point-based measurements, which makes it difficult to dynamically acquire information about soil salinity over large areas [6]. Moreover, most traditional techniques are limited to small-scale studies and cannot effectively monitor the spatial variability of salinity across extensive regions. These limitations highlight the need for more efficient and scalable monitoring systems.

In contrast, remote sensing technology offers the advantages of timeliness and wide coverage, making it a critical tool for monitoring soil salinization [7,8]. Remote sensing can detect changes in the Earth’s surface through the analysis of spectral characteristics, which are closely related to soil properties such as moisture content and salinity [6]. These spectral features form the basis for optical remote sensing methods, especially hyperspectral sensing, to estimate soil salinity [10]. Optical remote sensing systems rely on the reflection and absorption properties of various wavelengths of light to capture the spectral signature of the soil and vegetation, enabling the inversion of soil salinity [1,9,11,12,13]. However, optical remote sensing methods are highly sensitive to atmospheric conditions, such as cloud cover and precipitation, which can limit their effectiveness. Additionally, spectral overlaps between different surface types can cause ambiguities, leading to inaccurate salinity estimates [14].

Synthetic aperture radar (SAR) offers distinct advantages over optical methods, especially in arid and semi-arid regions [15]. As an active sensing technology, SAR is less affected by atmospheric conditions such as clouds and rain, providing continuous, all-weather, all-time observation capabilities [16]. This is particularly important for monitoring soil salinization, as optical sensors can be severely hindered by weather and lighting conditions. The key to SAR’s effectiveness in soil salinity monitoring lies in its interaction with the soil’s surface and subsurface layers. When SAR waves, which are in the microwave spectrum, impinge upon the soil, their interaction with the soil is influenced by its dielectric properties, which are highly sensitive to moisture content and salt levels [17,18,19]. Soil salinity influences the dielectric constant of the soil, which directly affects how radar waves scatter when they encounter the soil surface. The presence of salt increases the soil’s dielectric constant, leading to stronger backscatter signals from the radar [18]. This allows SAR to detect variations in soil salinity, even at depths below the surface, as microwave signals can penetrate the upper layers of the soil to a certain extent, depending on their frequency and the soil’s moisture content. This depth penetration capability is a significant advantage over optical methods, which only provide information from the soil surface [19]. Moreover, fully polarimetric SAR measures the backscattered signal at different polarizations (e.g., HH, HV, VH, VV), providing a richer dataset compared to single-polarization SAR [15]. This additional information helps to distinguish between different soil properties, such as roughness, moisture, and salinity, improving the accuracy of soil salinity estimates. The ability to capture multiple scattering mechanisms, such as surface scattering and volume scattering, further enhances SAR’s sensitivity to the variations in salt content across the landscape [20]. These combined features make SAR a powerful tool for monitoring soil salinity, enabling it to provide a more detailed and accurate understanding of the spatial distribution and temporal changes of salt concentrations over large areas.

Numerous studies have explored the use of feature space models for soil salinity monitoring, leveraging remote sensing spectral indices to quantify soil salinity levels [1,4,9,12,13,21]. Feature space refers to a multi-dimensional space constructed from different characteristics derived from remote sensing data, where each dimension represents a specific property or index related to the target material—in this case, soil salinity [9]. By combining multiple spectral bands or indices, researchers can map the distribution of soil salinity more effectively [11]. For instance, Wang et al. (2010) [1] proposed the NDVI–salinization index (NDVI-SI) feature space to analyze spatiotemporal changes in salinity in the Yutian Oasis. Additionally, Ding et al. (2014) [22] employed spectral unmixing techniques to construct multi-dimensional feature spaces, which helped in extracting information about salinized soils. These studies demonstrate that remote sensing-based feature space models are effective in quantifying the spatial distribution of surface salinity, providing valuable insights into the dynamics of soil salinization.

However, the majority of these studies have relied on optical remote sensing indices to construct feature spaces [23,24,25,26]. As mentioned earlier, while optical remote sensing has been widely applied in soil salinity estimation, it is susceptible to atmospheric interference and spectral overlap, leading to inaccuracies [27]. In contrast, radar remote sensing, particularly polarimetric SAR, offers unique advantages. In recent years, research combining radar and optical data has also shown promise. For example, Muhetaer et al. (2022) [28] demonstrated the potential of radar-based feature space models for soil salinity retrieval in arid regions. Despite these advances, the use of multi-polarization SAR data for soil salinity monitoring remains relatively underexplored, presenting significant opportunities for further research and development.

In this context, this study focuses on the Yutian Oasis, an area characterized by its vegetation and salinization status. Using SAR data from the Gaofen-3 satellite, along with field measurements, we constructed a radar feature space model for remote sensing monitoring of soil salinization in arid regions. The objective of this study was to explore the unique advantages of polarimetric radar data for soil salinity retrieval and to develop an effective, scalable method for monitoring soil salinization in arid areas. This research aims to provide new insights into the application of polarimetric SAR data for soil salinization monitoring, contributing to improved soil management and agricultural sustainability in regions affected by salinization.

## 2. Materials and Methods

### 2.1. Study Area

**Figure 1:**
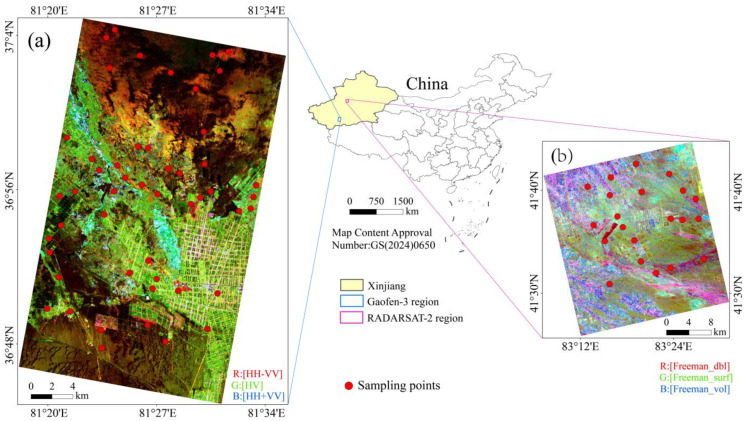
Study areas. (**a**) Field sampling points and Gaofen-3 imagery of primary study area (Yutian Oasis). (**b**) Field sampling points and RADARSAT-2 imagery of secondary study area (Weiku Oasis).

The Yutian Oasis (36°47′–37°06′ N, 81°08′–81°45′ E), located in southwestern Xinjiang, China, was the primary study area for model construction. As a typical oasis–desert transition zone, it features an arid continental climate with a complex mountain-basin geomorphology [29]. Elevation ranges from 1304 to 1639 m, with a multi-year average temperature of 12.4 °C. The region receives extremely low annual precipitation (14 mm) but has high evaporation (2500 mm) [30], making it heavily dependent on meltwater from mountain glaciers and groundwater recharge. The Keriya River, a seasonal river originating in the Kunlun Mountains, flows through the oasis before vanishing into the Taklamakan Desert. The study area covers 682.92 km^2^ (Figure 1a).

Soil salinization affects nearly 30% of irrigated land in the Yutian Oasis [3], primarily due to aridity, low rainfall, and irrigation with saline groundwater/meltwater. High evaporation concentrates dissolved salts in the soil, while shallow groundwater tables further accelerate salinization. This severely limits agricultural productivity and threatens the ecological balance, making the region a critical case for salinization monitoring.

To assess model generalizability, a secondary study area—the Weiku Oasis (Figure 1b, Weigan–Kuqa River Delta Oasis, 82°10′–83°05′ E, 41°06′–41°40′ N)—was selected. This region has a warm–temperate arid climate (multi-year average temperature: 11.6 °C; annual precipitation: 52 mm; annual evaporation: 2420 mm) and diverse soils (e.g., fluvo-aquic, saline, meadow soils) [31]. Like Yutian, it faces severe salinization challenges due to high evaporation and low rainfall, providing a suitable secondary validation site.

### 2.2. Remote Sensing Data and Preprocessing

This study utilized two types of remote sensing imagery: (1) Gaofen-3 fully polarimetric SAR data from the Yutian Oasis for model construction and (2) RADARSAT-2 fully polarimetric SAR data from the Weiku Oasis for model generalizability evaluation.

The Gaofen-3 (GF-3) satellite, launched on 10 August 2016, is China’s first domestically developed civilian C-band fully polarimetric synthetic aperture radar satellite. It has a high spatial resolution and diverse imaging modes, making it a critical SAR data source for remote sensing research in China. Its maximum resolution of 1 m significantly enhances the precision of Earth observations [32]. Since its deployment, Gaofen-3 data have been widely utilized in various fields, such as target detection, meteorological research, urban disaster prevention and mitigation, and post-disaster reconstruction [3,20,25,33,34]. As a domestically developed satellite, Gaofen-3 data are relatively easy to access at a low cost, facilitating its use by researchers and practitioners. The Gaofen-3 SAR data acquired on 4 May 2020 (same period with field sampling) were selected for use in this study. The parameters of these data are presented in Table 1.

RADARSAT-2, a commercially operated C-band polarimetric SAR satellite developed by the Canadian Space Agency (CSA), provides high-quality Earth observation data with flexible imaging modes and stable performance. Its fully polarimetric capability makes it suitable for cross-regional model validation. The RADARSAT-2 data used in this study were acquired on 23 April 2014 (same period with field sampling) under Fine Quad-Pol. Key specifications are provided in Table 1.

To ensure data accuracy and usability, the Gaofen-3 and RADARSAT-2 SAR imagery underwent comprehensive preprocessing, including radiometric calibration, speckle filtering, multi-looking, orthorectification, and geocoding [35].

### 2.3. Soil Sample Collection and Laboratory Analysis

Soil samples were collected from 30 April to 11 May to capture stable surface salinity conditions following winter accumulation and prior to significant summer leaching effects, while coinciding with early crop growth stages for salinity impact assessment [22]. In the primary Yutian Oasis study area, 50 representative sampling sites were strategically selected across agricultural core zones, oasis–desert transition areas, and visible salinization hotspots, guided by Ovitalmap and land-use maps to ensure coverage of varied soil textures and spatial salinization patterns, with all sites aligned to Gaofen-3 SAR image coverage. For model generalizability validation in the Weiku Oasis, 21 comparable sites were selected following the same sampling protocol to align with the salinization gradient and major land-use categories observed in the Yutian Oasis within the coverage area of RADARSAT-2 SAR imagery. At each 10 × 10 m plot, topsoil (0–10 cm) was collected using a 5-point composite method, with approximately 500 g subsamples stored in sealed bags after GPS documentation.

In the lab, the samples were air-dried, ground, and sieved to eliminate any impurities. Next, a 1:5 soil–water suspension was prepared, oscillated 200 times, and allowed to stand for 4 h to ensure thorough mixing. This was followed by filtration. Within a specific concentration range, the soluble salt content in a soil solution is positively correlated with its electrical conductivity. This relationship is commonly represented by a regression equation, which enables the conversion of electrical conductivity measurements into salinity values [36]. In this study, a METTLER TOLEDO^®^ FE38 conductivity meter (Mettler-Toledo GmbH, Greifensee, Switzerland) was used to measure the electrical conductivity of the soil solution. This instrument computes the soil salinity using either built-in empirical relationships or calibration curves. The descriptive statistics of the obtained soil conductivity data are presented in Table 2.

### 2.4. Methods

**Figure 2:**
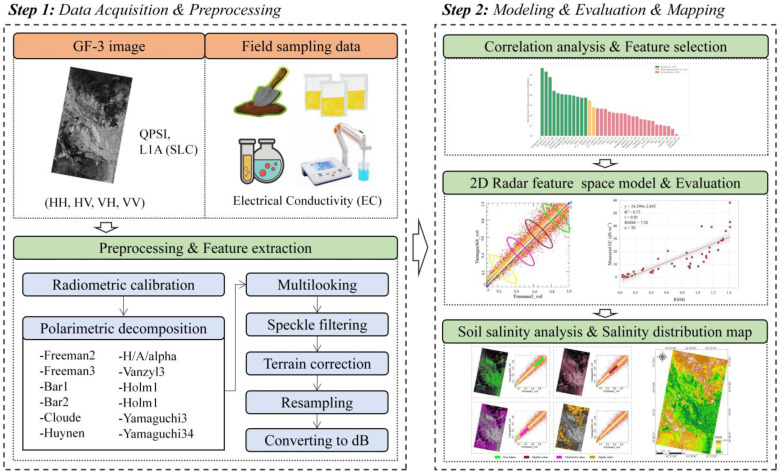
Workflow of this study.

In this study, based on fully polarimetric Gaofen-3 SAR data and field measurement data, we utilized polarimetric target decomposition techniques to extract various scattering components from the SAR imagery. Pearson correlation analysis and significance tests were then conducted to identify the two most relevant feature components. A two-dimensional feature space was constructed based on feature space theory, and subsequently, a radar salinization monitoring index (RSMI) model was developed using this feature space. The workflow of this research is illustrated in Figure 2.

#### 2.4.1. Polarimetric SAR (PolSAR) Target Decomposition

**Figure 3:**
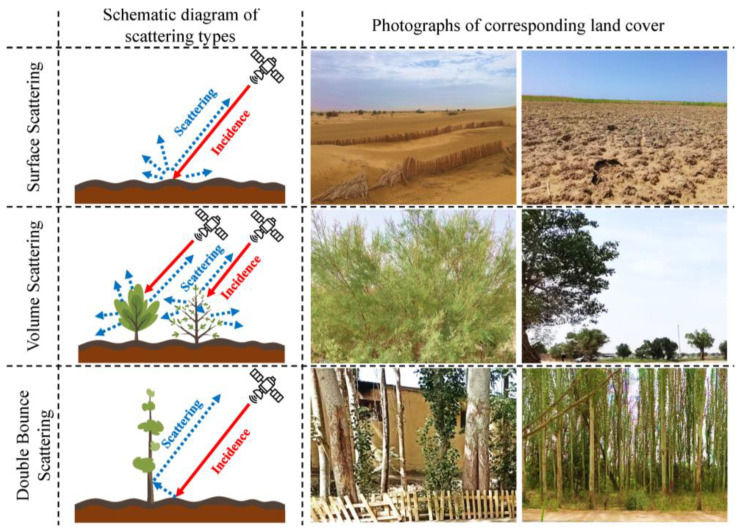
Scattering properties of SAR signal interactions with different ground targets.

Synthetic aperture radar (SAR) backscattering occurs when electromagnetic waves interact with target surfaces and part of the energy returns to the radar receiver. This process encompasses three primary scattering (Figure 3) mechanisms [37,38,39].

Polarimetric target decomposition is a technique for processing SAR data. It decomposes SAR signals into several fundamental scattering components, and each component represents a different physical scattering mechanism of the surface [40,41,42]. These components help explain and interpret the physical significance of SAR data. The core purpose of target decomposition methods is to decompose the scattering matrix, covariance matrix, or coherence matrix into a linear combination of several independent matrices, with each independent matrix corresponding to an independent element of the target and representing its associated physical mechanism [26]. This decomposition strategy not only facilitates efficient extraction and identification of the target’s scattering characteristics but also provides deeper insights into the target’s physical properties.

Target decomposition methods are primarily classified into two types based on the scattering characteristics of the target: coherent target decomposition and incoherent target decomposition [43]. Coherent target decomposition aims to decompose the scattering matrix ***S***, which is obtained from radar measurements, into the sum of the scattering responses of several simple objects. Common coherent decomposition methods include Pauli decomposition, Cameron decomposition, symmetric scattering characterization method (SSCM) decomposition and sphere–diplane–helix (SDH) decomposition [44,45]. In contrast, incoherent target decomposition seeks to decompose the covariance matrix *C* or coherence matrix *T* into combinations of second-order descriptors corresponding to simple or standard objects, thus providing a more intuitive physical interpretation. Incoherent decomposition methods include Freeman–Durden decomposition, Cloude decomposition, and Huynen decomposition [46,47].

The polarimetric scattering matrix is a second-order complex matrix, generally represented as:

(1) $$S=\left[\begin{array}{cc} S_{HH} & S_{HV}\\ S_{VH} & S_{VV} \end{array}\right],\;$$

where *S**_ij_*** represents the backscattering coefficients for the different polarization modes.

The covariance matrix *C* can be expressed as:

(2) $$C=\left[\begin{array}{ccc} \left\langle {\left|S_{HH}\right|}^{2}\right\rangle & \left\langle S_{HH}S_{HV}^{*}\right\rangle & \left\langle S_{HH}S_{VV}^{*}\right\rangle \\ \left\langle S_{HV}S_{HH}^{*}\right\rangle & \left\langle {\left|S_{HV}\right|}^{2}\right\rangle & \left\langle S_{HV}S_{VV}^{*}\right\rangle \\ \left\langle S_{VV}S_{HH}^{*}\right\rangle & \left\langle S_{VV}S_{HV}^{*}\right\rangle & \left\langle {\left|S_{VV}\right|}^{2}\right\rangle \end{array}\right],\;$$

where $\left\langle \cdot \right\rangle$ represents the mathematical expectation (usually the average of multiple observations or samples). The superscript * denotes the conjugate. For example, $S_{HH}^{*}$ is the complex conjugate of $S_{HH}$.

The coherence matrix *T* can be defined through the elements of the scattering matrix as:

(3) $$T=\left[\begin{array}{cccc} S_{HH}S_{HH}^{*} & S_{HH}S_{HV}^{*} & S_{HH}S_{VH}^{*} & S_{HH}S_{VV}^{*}\\ S_{HV}S_{HH}^{*} & S_{HV}S_{HV}^{*} & S_{HV}S_{VH}^{*} & S_{HV}S_{VV}^{*}\\ S_{VH}S_{HH}^{*} & S_{VH}S_{HV}^{*} & S_{VH}S_{VH}^{*} & S_{VH}S_{VV}^{*}\\ S_{VV}S_{HH}^{*} & S_{VV}S_{HV}^{*} & S_{VV}S_{VH}^{*} & S_{VV}S_{VV}^{*} \end{array}\right],\;$$

where $S_{HH}S_{HH}^{*}$ represents the self-coherence of the horizontal-polarized channel. It reflects the stability and intensity of the horizontally-polarized echo signal. In the coherence matrix, the diagonal element is used to measure the energy of the horizontal polarization itself.

Based on these matrices, there are certain differences in the calculation of various polarimetric decomposition components [41]. For example, the Freeman decomposition utilizes the covariance matrix or scatter matrix for decomposition. The decomposition equations divide the elements of the covariance or correlation matrix into three primary components (surface scattering, volume scattering, and double-bounce scattering):

(4) $$C_{total}=C_{surf}+C_{dbl}+C_{vol},$$

where the _vol represents volume scattering, _surf denotes surface scattering, and _dbl indicates the double-bounce scattering component.

The Van Zyl decomposition also uses the coherence matrix *T*, separates the radar backscatter into volume, surface, and double-bounce components, similar to Freeman’s but with a focus on the physical understanding of scattering mechanisms:

(5) $$T_{total}=T_{suf}+T_{dbl}+T_{vol}.$$

The Yamaguchi decomposition is an extension of the Freeman and Van Zyl decompositions, and is particularly useful for analyzing data from high-resolution radar systems. It uses a set of components in the polarimetric scattering matrix, with the following equations for the various components:

(6) $$S=\left[S_{1},S_{2},S_{3},S_{4}\right],$$

where *S*_1_ is surface scattering, *S*_2_ is double-bounce scattering, *S*_3_ is volume scattering, and *S*_4_ is complex (helix) scattering from combinations of the above mechanisms.

The Cloude decomposition is based on the Eigenvalue decomposition of the coherence matrix, and it focuses on entropy, anisotropy, and alpha angles to characterize the scattering process, where the components are:

(7) $$M=V\cdot D\cdot V^{T},$$

where *M* is the scattering matrix, *V* is the matrix of eigenvectors, and *D* is the diagonal matrix of eigenvalues. The Cloude parameters (entropy, anisotropy, alpha) are derived from these eigenvalues:

Entropy (*H*), measures the disorder in the scattering process:

(8) $$H=-\sum_{i=1}^{n}\;\lambda_{i}\log\left(\lambda_{i}\right).$$

where $\lambda_{i}$ are the eigenvalues. A higher entropy value indicates a more random scattering process, suggesting a more complex scene with multiple scattering mechanisms contributing equally.

Anisotropy (*A*), measures the directional coherence of the scattering:

(9) $$A=\frac{\lambda_{max}-\lambda_{min}}{\lambda_{max}+\lambda_{min}}.$$

where $\lambda_{max}$ and $\lambda_{min}$ are the maximum and minimum eigenvalues, respectively.

Alpha (*α*), characterizes the dominant scattering mechanism’s orientation.

H/A/Alpha is a generalization of Cloud’s entropy method, where *H*, *A*, and *α* (Alpha) are calculated based on eigenvalues derived from the scattering matrix [48]. Its calculation process is similar to Cloud’s method, but this decomposition provides a more direct and specific interpretation of scattering using entropy, anisotropy, and alpha angle.

To maximize the potential of fully polarimetric Gaofen-3 data, in this study, we employed 12 polarimetric decomposition methods, namely, the Freeman2, Freeman3, Bar1, Bar2, Cloude, H/A/Alpha, Holm1, Holm2, Huynen, Van Zyl3, Yamaguchi3, and Yamaguchi4 methods [48,49,50,51,52,53,54,55,56,57,58,59,60], extracting a total of 36 feature components as shown in Table 3.

**Figure 4:**
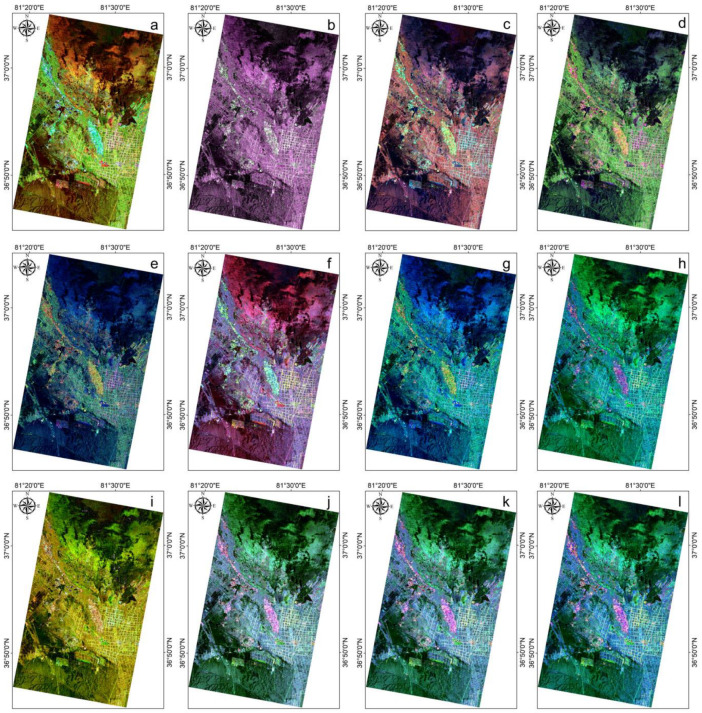
Different polarization target decomposition methods of Gaofen-3 radar imagery with RGB, including: (**a**) Freeman3 decomposition, (**b**) Freeman2 decomposition, (**c**) Bar1 decomposition, (**d**) Bar2 decomposition, (**e**) Cloude decomposition, (**f**) H/A/Alpha, (**g**) Holm1 decomposition, (**h**) Holm2 decomposition, (**i**) Huynen decomposition, (**j**) Van Zyl3 decomposition, (**k**) Yamaguchi3 decomposition, and (**l**) Yamaguchi4 decomposition.

The results of the polarimetric decomposition are presented in the form of RGB band combinations of polarimetric feature components (Figure 4).

As shown in Figure 4, the decomposition methods exhibit distinct scattering characteristics that are consistent with field observation. Freeman3 (Figure 4b) demonstrates superior information content compared to Freeman2 (Figure 4a), evidenced by its broader color range that better represents the balance between fundamental scattering components (surface scattering, volume scattering, and double-bounce scattering) across heterogeneous landscapes. While the three-component decompositions (Figure 4c–k) show a generally similar intensity distribution. The four-component Yamaguchi4 decomposition shows particular advantages, with its unique helix scattering component providing improved detection of anthropogenic features and demonstrating strong agreement with field measurements. The scattering mechanisms show characteristic responses: volume scattering dominates vegetated areas, surface scattering prevails in bare soils, and double-bounce scattering is pronounced in urban environments, all consistent with our field surveys.

#### 2.4.2. Correlation Analysis and Feature Selection

PolSAR polarization features reflect inherent scattering mechanisms of various terrain types, which are critical for terrain classification and earth observation applications. However, the extracted parameters often contain redundant information and noise, and not all parameters are suitable for soil salinity retrieval [62,63]. Although target decomposition methods can extract multiple polarimetric features, using all of the extracted features as the input in the retrieval process would significantly increase computational costs. Therefore, selecting appropriate features during the retrieval process is key to improving the accuracy. In light of this, the objective of this study was to identify and select features that are significant contributors to the model accuracy during the retrieval process so as to optimize the computational efficiency.

**Figure 5:**
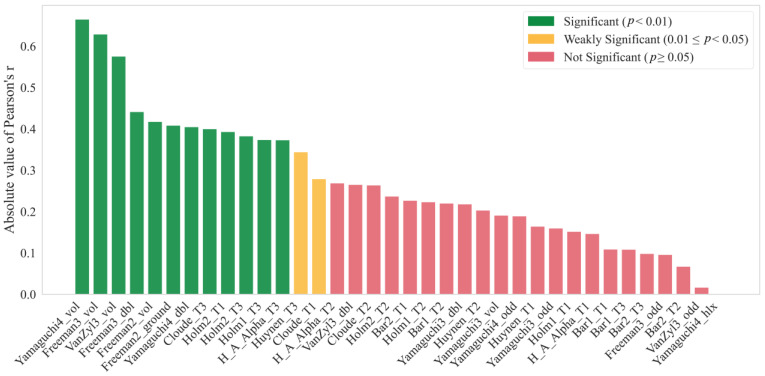
Correlation coefficients of polarized decomposition components with EC, ordered by absolute value (Note: _ground, _odd, and _T1 are surface scattering, _vol and _T2 are volume scattering, _dbl and _T3 are double-bounce scattering, and _hlx is helix scattering).

To achieve this objective, Pearson correlation coefficients (r) were used to quantify the correlations between 36 polarimetric features and the actual soil electrical conductivity, which is a metric of the actual soil salinity. The Pearson correlation coefficient (r) measures the linear relationship between two variables, ranging from −1 to +1. The sign indicates the direction of association (positive/negative), while the absolute value represents the strength of the relationship [64]. The correlations between these features and the measured soil conductivity are shown in Figure 5.

Two features, namely Yamaguchi4_vol and Freeman3_vol, were identified as having the highest correlations with the soil salinity, with correlation coefficients of −0.67 (*p* < 0.01) and −0.63 (*p* < 0.01), respectively. They were selected as two key features to construct a two-dimensional feature space, improving the precision and reliability of the salinity retrieval model and enhancing the efficiency and accuracy of the soil salinization monitoring.

#### 2.4.3. Normalization of Feature Parameters

To eliminate the effects of differences in the magnitudes of the features, normalization was applied to Yamaguchi4_vol and Freeman3_vol using the following formulas:

(10) $$\mathrm{Yamaguchi}4\text{\_}\mathrm{vol}=\frac{\mathrm{Yamaguchi}4\text{\_}\mathrm{vol}-{\mathrm{Yamaguchi}4\text{\_}\mathrm{vol}}_{min}}{{\mathrm{Yamaguchi}4\text{\_}\mathrm{vol}}_{min}-{\mathrm{Yamaguchi}4\text{\_}\mathrm{vol}}_{max}},$$

(11) $$\mathrm{Freeman}3\text{\_}\mathrm{vol}=\frac{\mathrm{Freeman}3\text{\_}\mathrm{vol}-{\mathrm{Freeman}3\text{\_}\mathrm{vol}}_{min}}{{\mathrm{Freeman}3\text{\_}\mathrm{vol}}_{min}-{\mathrm{Freeman}3\text{\_}\mathrm{vol}}_{max}},$$

where ${\mathrm{Yamaguchi}4\text{\_}\mathrm{vol}}_{max}$ and ${\mathrm{Yamaguchi}4\text{\_}\mathrm{vol}}_{min}$ are the maximum and minimum values of Yamaguchi4_vol, respectively; and ${\mathrm{Freeman}3\text{\_}\mathrm{vol}}_{max}$ and ${\mathrm{Freeman}3\text{\_}\mathrm{vol}}_{min}$ are the maximum and minimum values of Freeman3_vol, respectively.

#### 2.4.4. RSMI Model

A feature space is constructed using multiple bands or indices to represent surface parameters, and the number of parameters and bands is typically two or more [11]. In the feature space, image pixels are geometrically distributed according to a certain geometric pattern. Different types of ground objects appear in distinct locations, while ground objects with the same or similar attributes exhibit clustered distributions in the feature space [65]. Based on this principle, the soils with different degrees of salinization in the study area can be effectively distinguished.

**Figure 6:**
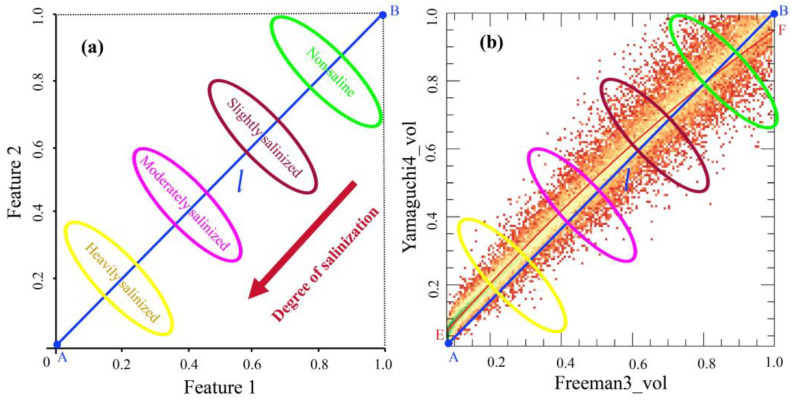
(**a**) Conceptual framework of feature space approach. (**b**) Mathematical derivation of RSMI index, demonstrating relationship between Euclidean distance (*l*) and salinity levels. Color gradient represents different salinity levels. *EF* represents the trendline (fitted curve) within the feature space; *A* denotes the origin of the coordinate system (0, 0); and *B* signifies an arbitrary point (1, 1). *l* denotes the length of the curve EF (simplified to the line AB), which corresponds to the distance from point *A* to point *B*.

After normalizing the polarimetric feature components, a two-dimensional feature space was constructed based on feature space theory, with Freeman3_vol as the horizontal axis and Yamaguchi4_vol as the vertical axis (Figure 6).

As can be seen from Figure 6b, there is a nonlinear relationship between Yamaguchi4_vol and Freeman3_vol. According to the study conducted by Verstraete et al. (1996) [9], the trajectory curve of the soil salinization (fitted curve *EF* in Figure 6b) between the two features can be simplified into a straight line *l*. Verstraete et al. (1996) [9] also pointed out that in the feature space, the distance perpendicular to the feature trajectory line ***l*** can effectively distinguish between soils with different degrees of salinization. Therefore, the distance from any point in the feature space to the coordinate origin *A* (0, 0) can represent the soil salinity at that point. Specifically, soil salinity is inversely proportional to the distance from the origin: the farther the distance from the origin, the lower the soil salinity and the milder the salinization. Conversely, the shorter the distance to the origin is, the higher the soil salinity is and the more severe the salinization is. Within the feature space, the distance *l* from any point (such as point *B* (1,1)) to point *A* can be obtained using the following formula:

(12) $$l=\sqrt{{\left(\mathrm{Feature}1\right)}^{2}{+(\mathrm{Feature}2)}^{2}}.$$

Accordingly, the RSMI model is established by the following formula:

(13) $$RSMI=l=\sqrt{{\left(\mathrm{Yamaguchi}4\text{\_}\mathrm{vol}\right)}^{2}{+(\mathrm{Freeman}3\text{\_}\mathrm{vol})}^{2}}.$$

## 3. Results

### 3.1. Validation of the Accuracy of the RSMI Model

**Figure 7:**
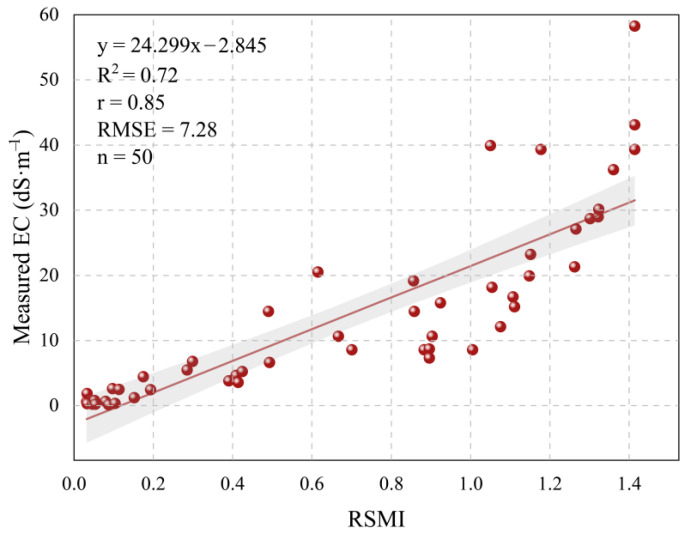
Fitting of measured EC and simulated values of RSMI model.

To verify the practicality and reliability of the RSMI model, linear regression analysis was conducted between the soil electrical conductivity data for 50 soil samples and the RSMI values extracted at the coordinates of the sampling sites (Figure 7). The resulting fitting equation is y = 24.299x − 2.845, where x is the model-predicted RSMI value, and y is the soil electrical conductivity measured in the laboratory. The regression analysis revealed that there is a strong correlation between the RSMI-predicted values and the actual soil conductivity (r = 0.85), with a coefficient of determination (R^2^) of 0.72 and a root mean square error (RMSE) of 7.28 dS/m. These results indicate that the RSMI model effectively reflects the actual spatial distribution of the different salinization levels in the study area and can be used for rapid monitoring of soil salinization.

### 3.2. Analysis of Spatial Pattern of Salinization Using the Yamaguchi4_vol-Freeman3_vol Feature Space

**Figure 8:**
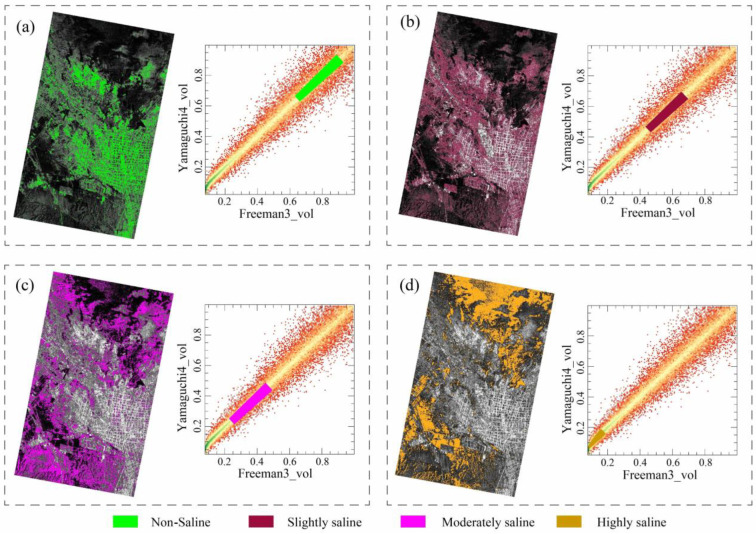
Distribution of different salinized soils in Freeman3_vol-Yamaguchi4_vol feature space. (**a**) Non-salinized soils, (**b**) slightly salinized soils, (**c**) moderately salinized soils, and (**d**) severely salinized soils.

The Yamaguchi4_vol-Freeman3_vol feature space was used to analyze the soil salinization status in the study area. As shown in Figure 8, in the two-dimensional feature space, the degree of soil salinization gradually decreases as the Yamaguchi4_vol and Freeman3_vol components increase, indicating the existence of negative correlations between the soil salinization and the two components. In the study area, at locations with dense crop growth and good vegetation conditions, the intensities of the volumetric scattering components are higher (Figure 3), while the soil salinity is lower at these sites. Conversely, at locations with poor soil water retention and higher salinization levels, the intensity of the volumetric scattering component decreases as the vegetation coverage decreases. The spatial distribution of the salinized soils exhibits a concentric pattern, transitioning from slightly salinized areas to moderately salinized areas and then to heavily salinized areas. This spatial pattern of soil salinization observed in the Yamaguchi4_vol-Freeman3_vol feature space is consistent with the field observations and sample analyses.

### 3.3. RSMI Model-Based Quantitative Retrieval of Soil Salinity

**Figure 9:**
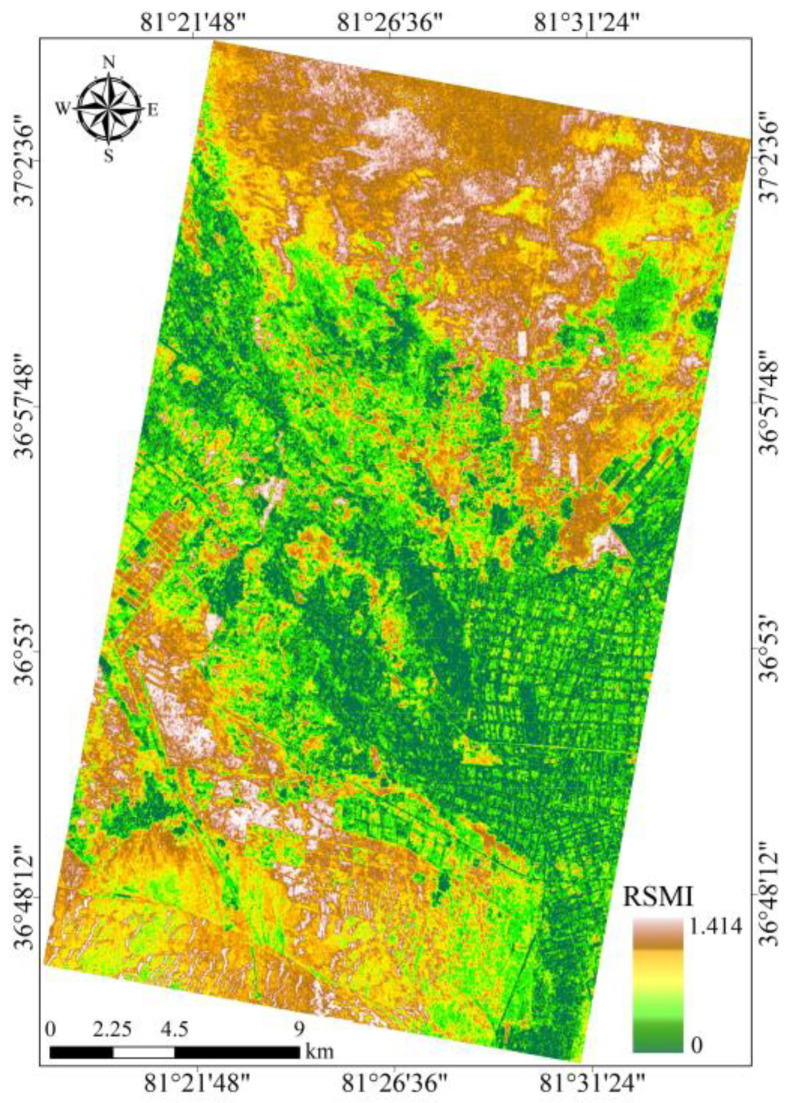
Spatial distribution pattern of soil salinity in study area.

RSMI model-based retrieval of the soil salinity was performed for the entire study area (Figure 9). In Figure 9, the variation in the depth of the color directly reflects the distribution characteristics of the soil salinity: the dark brown areas correspond to high-risk regions with elevated soil salinity, while the dark green areas are regions with lower soil salinity and relatively mild salinization. It can be clearly seen from Figure 9 that the areas with slightly soil salinization are mainly concentrated in the central parts of the oasis and its surroundings where the water conditions and vegetation coverage are typically better. In contrast, the areas with more severe soil salinization tend to be distributed in the low-lying outskirts of the oasis, where higher water evaporation and poor drainage conditions lead to salt accumulation.

## 4. Discussion

### 4.1. RSMI Model Generalizability Analysis

To validate the generalizability of the proposed RSMI model in similar arid/semi-arid environments, we selected the Weiku Oasis in Xinjiang as a secondary study area (Figure 1b). This region shares comparable climatic conditions, soil types, and salinization mechanisms with the Yutian Oasis (primary study area). The evaluation was conducted using RADARSAT-2 data and ground truth, with consistent sensor configurations and sampling protocols as the Yutian dataset.

**Figure 10:**
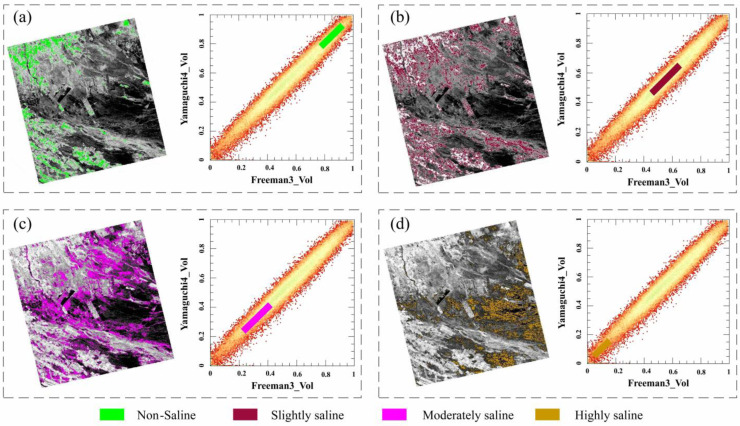
Distribution of different salinization levels in Freeman3_vol-Yamaguchi4_vol feature space for Weiku Oasis. (**a**) Non-salinized soils, (**b**) slightly salinized soils, (**c**) moderately salinized soils, and (**d**) severely salinized soils.

The same methodology was applied to decompose RADARSAT-2 data into Freeman3_vol and Yamaguchi4_vol components, reconstructing the RSMI 2D feature space (Figure 10). The distribution of salinized soils in this two-dimensional space (Figure 10a–d) demonstrates that the RSMI model effectively separates different salinity levels (e.g., mild/moderate/severe) in the Weiku Oasis, confirming its adaptability to new spatial contexts.

**Figure 11:**
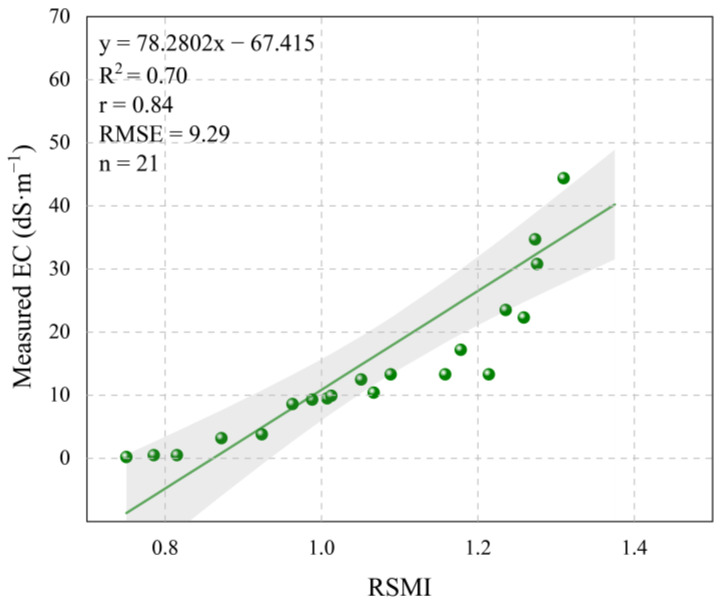
Performance evaluation of RSMI model in Weiku Oasis.

The accuracy validation of the RSMI model in the Weiku Oasis (Figure 11) demonstrates its robust performance in detecting soil salinization across varying degrees of severity. The linear fitting between measured EC and RSMI-simulated values yielded a strong correlation (r = 0.84), with a coefficient of determination (R^2^ = 0.70) and an RMSE of 9.29 dS/m. The fitted equation (y = 78.2802x − 67.415) indicates a systematic relationship between model predictions and field measurements, though with a steeper slope compared to the primary study area (Yutian Oasis).

When compared to the Yutian Oasis results (Figure 7; y = 24.299x − 2.845, R^2^ = 0.72, RMSE = 7.28 dS/m), the Weiku Oasis validation (Figure 11) exhibits two key differences: (1) The slope of the fitting equation in Weiku (78.28) is significantly steeper than in Yutian (24.30), suggesting higher sensitivity of RSMI to EC variations in Weiku. This could arise from differences in soil composition (e.g., higher gypsum content) or radar backscatter responses under distinct surface roughness conditions; (2) The slightly higher RMSE in Weiku Oasis (9.29 vs. 7.28 dS/m) may reflect regional-specific challenges, such as fragmented saline patches or localized irrigation effects. Nevertheless, the comparable R^2^ values (0.70 vs. 0.72) confirm consistent explanatory power across both regions.

**Figure 12:**
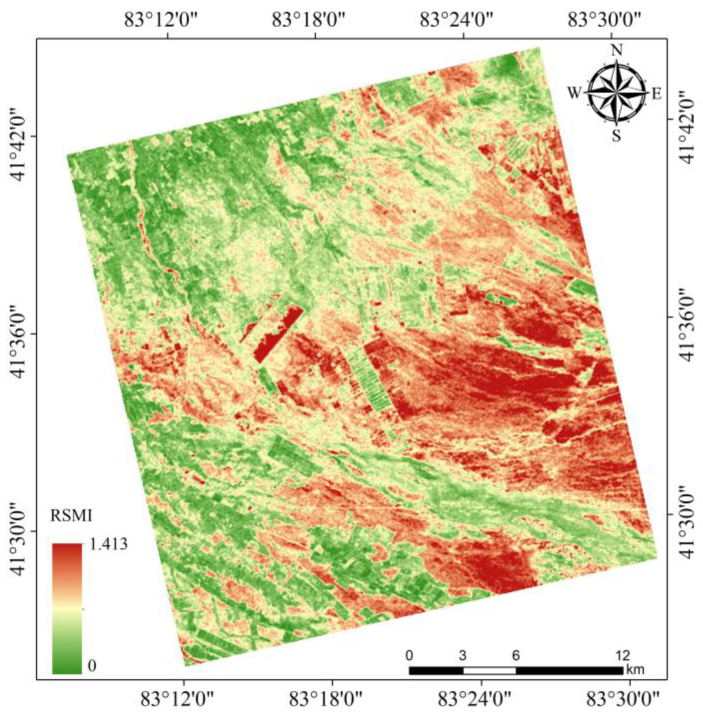
Spatial distribution pattern of soil salinity in Weiku Oasis.

The derived salinity map (Figure 12) reveals that salinization in Weiku Oasis primarily distributes along irrigation canals and low-lying depressions (41°26′ N–41°45′ N, 83°08′ E–83°32′ E), with severe salinization (red/orange areas) concentrated in the central-eastern sector where drainage is poor. Moderate salinity (yellow/green) forms transitional zones adjacent to these hotspots, while non-saline areas (blue/green) dominate elevated terrain in the northwest. This spatial pattern mirrors Weiku Oasis’s salt accumulation mechanisms: saline groundwater rise in lowlands (W–E orientation) and secondary salinization along canal networks (N–S trending). The distribution aligns with the region’s arid hydrology, where capillary action in clay-rich depressions drives salt enrichment. Such consistency with Yutian Oasis’s hydrogeomorphic drivers (e.g., elevation-controlled waterlogging, anthropogenic water diversion) confirms the model’s capacity to universalize salinization processes across arid basins.

The RSMI model’s ability to maintain relatively high accuracy in Weiku, despite differing regression parameters, underscores its adaptability to similar arid/semi-arid environments. The alignment of salinity distribution patterns with hydrogeomorphic drivers (e.g., irrigation canals, low-lying areas; Figure 12) further validates the model’s capacity to universalize salinization mechanisms. Minor discrepancies in slope and error metrics likely stem from site-specific factors (e.g., soil texture, microclimate) rather than model limitations, emphasizing the need for localized calibration when extending RSMI to new regions.

The Weiku Oasis validation corroborates the RSMI model’s generalizability for rapid salinization monitoring in arid landscapes, with consistent performance across diverse yet climatically analogous regions. Future work should explore scaling adjustments to harmonize slope variations while retaining the model’s core robustness.

### 4.2. Advantages, Limitations and Future Work

Soil salinization in the Yutian Oasis is a persistent and complex issue, exacerbated by the region’s arid climate and agricultural practices [29]. Monitoring this phenomenon with high accuracy is crucial for effective land management and sustainable agricultural practices. Traditional optical remote sensing methods have been widely applied for salinity monitoring, leveraging their rich spectral data to identify soil properties [6]. However, optical techniques are often limited by environmental factors such as cloud cover, vegetation, and lighting conditions, particularly in arid regions like Yutian Oasis [66]. In contrast, radar remote sensing offers distinct advantages, such as the ability to penetrate clouds, vegetation canopy, and subsoil profiles and its independence from lighting conditions, making it an ideal tool for monitoring soil salinization in such environments [45].

In this study, we have demonstrated the potential of radar remote sensing, specifically polarimetric SAR data, for more efficient and rapid soil salinity retrieval. Unlike traditional optical models, radar-based models for soil salinity monitoring remain relatively underexplored, with few studies employing radar data for feature space construction. One such study by Muhetaer et al. (2022) [28] showed promising results, using ALOS PALSAR-2 data to construct soil salinity retrieval models. However, their models exhibited relatively low correlation with field measurements (0.61–0.63), indicating room for improvement. In contrast, our proposed Radar Salinity Monitoring Index (RSMI) model, based on the Yamaguchi4_vol-Freeman3_vol feature space, achieved a higher correlation (r = 0.85) with soil salinity and yielded a strong linear fit with field data (R^2^ = 0.72, RMSE = 7.28 dS/m). These results highlight the potential of radar data for precise soil salinity retrieval, especially in regions where optical methods struggle.

The relationship between radar characteristics and soil salinity is complex, with different scattering components providing distinct insights into the internal structure of the soil. Our correlation analysis revealed that several scattering components exhibited relatively strong correlations with soil conductivity, particularly volume scattering, where the correlation was more pronounced (Table 4). Among the radar polarization decomposition components, Yamaguchi4_vol (volume scattering) showed the highest correlation (r = −0.67) with soil salinity. Similarly, Freeman3_vol demonstrated a notable, though somewhat weaker, correlation (r = −0.63). These results suggest that volume scattering is particularly sensitive to changes in soil salinity, likely due to its ability to detect variations in vegetation and soil structure. In contrast, surface scattering components, such as Cloude_T1 and Bar1_T1, showed weaker correlations but still contributed valuable information on the interaction between radar waves and salinized soils.

The advantage of volume scattering in detecting soil salinity becomes more apparent when compared with other scattering types. In Yamaguchi decomposition, Yamaguchi4_vol showed a stronger correlation with soil salinity than both Yamaguchi4_dbl (double-bounce scattering) and Yamaguchi4_odd (surface scattering). Similarly, Freeman3_vol exhibited a more significant relationship with soil salinity than its double-bounce counterparts. Other volume scattering components, such as Van Zyl3_vol, also demonstrated higher correlations, though weaker (r = −0.58) compared to Yamaguchi4_vol and Freeman3_vol. While surface scattering components generally displayed weaker correlations, they still provided valuable supplementary insights, helping to further understand the radar wave scattering mechanisms influenced by soil salinity changes.

The feature space created by combining Yamaguchi4_vol and Freeman3_vol proved to be effective for classifying soil salinity levels. This two-dimensional feature space allowed for clear differentiation between soil samples with varying salinity levels, with low-salinity soils forming distinct clusters at higher feature values. As salinization increased, the soil samples shifted toward lower feature values, reflecting the increased backscattering due to changes in the soil’s surface roughness and composition, which are influenced by the presence of salt. This clear spatial differentiation of soil salinity levels provides a robust framework for monitoring salinization and identifying areas at risk of severe salinity. The ability to visualize these patterns facilitates the development of targeted land management strategies, crucial for mitigating the effects of salinization.

While radar remote sensing holds great promise, it is not without limitations. The accuracy of radar-based salinity retrieval can be affected by factors such as soil moisture, surface roughness, and speckle noise [16]. For example, variations in soil moisture can alter the dielectric properties of the soil, leading to misinterpretations of radar signals and affecting salinity estimates [38]. Speckle noise, inherent in SAR data, further complicates the retrieval process by reducing the quality and reliability of the data [67]. These challenges highlight the need for further optimization in radar data processing and model calibration to mitigate these effects.

While demonstrating strong potential, radar-based salinity retrieval faces several challenges. First, inherent limitations of SAR data including soil moisture variations, surface roughness effects, and speckle noise can influence accuracy [46,68]. Second, while offering advantages over optical methods in cloud penetration and sub-surface detection, SAR alone cannot match optical data’s spectral richness for vegetation and soil texture characterization [69]. Most importantly for this study, C-band’s vegetation sensitivity requires special consideration though our results show effective application in arid regions due to: (1) strategic dry-season acquisition minimizing vegetation dynamics; and (2) comprehensive field validation confirming sparse ground conditions.

These findings suggest a path forward for more comprehensive salinity monitoring through synergistic integration of multi-source remote sensing data. The complementary strengths of SAR (physical structure detection), optical (spectral information), and other emerging data sources can be leveraged through hybrid modeling approaches. Particularly promising are: (1) multi-sensor data fusion combining C-band SAR with L-band ALOS PALSAR-2/NISAR and optical datasets (Sentinel-2, Landsat 9) to address vegetation interference while enhancing spectral discrimination; (2) incorporation of ancillary geospatial data including soil moisture (SMAP), topography (LiDAR/DEM), and meteorological records to improve model robustness; and (3) advanced machine learning techniques, particularly physics-informed deep learning, to disentangle complex vegetation-salinity interactions and refine nonlinear relationships between multi-source features and salinity levels [54,55]. Such integrated approaches could extend reliable salinity monitoring to more vegetated areas while maintaining accuracy in arid regions, with the current study’s feature space framework serving as a foundation for these more comprehensive monitoring systems.

## 5. Conclusions

In this study, we focused on the Yutian Oasis, developed a soil salinity retrieval model based on a two-dimensional feature space that was constructed via polarimetric target decomposition of remote sensing data from the fully polarimetric Gaofen-3 radar in relation to field measurement data, and applied the model for quantitative retrieval of the soil salinity in this region. The conclusions of this study are summarized below.

(1) In this study, through correlation analysis between 36 polarimetric components and the actual soil electrical conductivity, we successfully identified two key features, Yamaguchi4_vol and Freeman3_vol, which were significantly correlated (both *p* < 0.001) with the actual soil electrical conductivity, with correlation coefficients of −0.67 and −0.63, respectively. These results confirm the potential of polarimetric features in soil salinity retrieval.
(2) The RSMI model proposed in this study achieved a higher correlation (r = 0.85) with the soil surface salinity. The linear fit between the values obtained using the RSMI model and the measured soil electrical conductivity values yielded an R^2^ value of 0.72 and an RMSE of 7.28 dS/m, validating the effectiveness and reliability of the RSMI model in monitoring the different degrees of soil salinization. Furthermore, when applied to the Weiku Oasis using RADARSAT-2 data, the model maintained good performance (R^2^ = 0.70, RMSE = 9.29 dS/m), demonstrating its potential for regional application in similar arid environments.
(3) In the study area, the degree of soil salinization exhibits a spatial pattern of gradually increasing from the center of the oasis toward its periphery. This pattern is consistent with field observations, providing an intuitive radar remote sensing interpretation of the spatial distribution characteristics of the soil salinization in the Yutian Oasis.

In summary, the results of this study demonstrate that target decomposition and feature components can effectively retrieve surface soil salinity information in this region. Considering the unique geographic environments of arid regions, this method facilitates quantitative analysis and monitoring of soil salinization in such areas. The successful application in both Yutian and Weiku Oases suggests the model’s robustness across different but similar arid ecosystems. Moreover, the findings of this study provide new perspectives and methods for quantitatively retrieving soil salinity information using fully polarimetric radar remote sensing data.

## Figures and Tables

**Table 1:** Parameters of Gaofen-3 imagery.

Parameter Types	Gaofen-3	RADARSAT-2
Product Format	GeoTIFF	GeoTIFF
Projection Method	UTM	UTM (WGS84)
Imaging Mode	QPSI (Quad-Pol Single-Look)	Fine Quad-Pol
Polarization Mode	Quad-Pol (VH, HV, VV, HH)	Quad-Pol (HH, HV, VH, VV)
Processing Level	LEVEL 1.1	SLC (Single Look Complex)
Frequency	C-band (1.2 GHz)	C-band (5.405 GHz)
Resolution	2.2 × 5.5 m (Range × Azimuth)	5.5 m × 4.8 m (Range × Azimuth)
Incidence Angle	35.3°	41.05°
Antenna Look Direction	Right-Looking	Right-Looking
Orbit Direction	Descending	Descending

**Table 2:** Descriptive statistics of soil electrical conductivity.

Minimum	Maximum	Mean	Median	Standard Deviation	Coefficient of Variation
0.137 dS/m	58.26 dS/m	13.98 dS/m	8.63 dS/m	13.72 dS/m	98.14%

**Table 3:** Polarization decomposition feature components extracted from Gaofen-3 data.

Decomposition Method	Component Number	Decomposition Component	Reference
Freeman2	2	Freeman2_ground, Freeman2_vol.	[53]
Freeman3	3	Freeman3_odd, Freeman3_dbl, Freeman3_ vol.	[53]
Bar1	3	Bar1_T1, Bar1_T2, Bar1_T3.	[61]
Bar2	3	Bar2_T1, Bar2_T2, Bar2_T3.	[61]
Cloude	3	Cloude_T1, Cloude_T2, Cloude_T3.	[52]
H/A/Alpha	3	H_A_Alpha_T1, H_A_Alpha_T2, H_A_Alpha_T3.	[48]
Holm1	3	Holm1_T1, Holm1_T2, Holm1_T3.	[61]
Holm2	3	Holm2_T1, Holm2_T2, Holm2_T3.	[61]
Huynen	3	Huynen_T1, Huynen_T2, Huynen_T3.	[49]
Van Zyl3	3	Van Zyl3_odd, Van Zyl3_dbl, Van Zyl3_vol.	[57]
Yamaguchi3	3	Yamaguchi3_odd, Yamaguchi3_dbl, Yamaguchi3_vol.	[54]
Yamaguchi4	4	Yamaguchi4_ odd, Yamaguchi4_dbl, Yamaguchi4_vol, Yamaguchi4_hlx.	[54]

Note: _ground, _odd, and _T1 denote surface scattering; _dbl and _T2 indicate double-bounce scattering; _vol and _T3 represent volume scattering; and _hlx signifies helix scattering.

**Table 4:** Correlation coefficient of radar target polarization decomposition components and soil salt content.

Variables	r	Variables	r	Variables	r
Yamaguchi4_vol	−0.67 **	Huynen_T3	0.34 *	Yamaguchi4_odd	−0.19
Freeman3_vol	−0.63 **	Cloude_T1	−0.28 *	Huynen_T1	−0.16
VanZyl3_vol	−0.58 **	H_A_Alpha_T2	0.27	Yamaguchi3_odd	−0.16
Freeman3_dbl	−0.44 **	VanZyl3_dbl	−0.27	Holm1_T1	−0.15
Freeman2_vol	−0.42 **	Cloude_T2	0.26	H_A_Alpha_T1	−0.15
Yamaguchi4_dbl	−0.41 **	Holm2_T2	0.24	Bar1_T1	−0.11
Freeman2_ground	0.41 **	Bar2_T1	−0.23	Bar1_T3	0.11
Cloude_T3	0.40 **	Holm1_T2	0.22	Bar2_T3	0.10
Holm2_T1	−0.39 **	Bar1_T2	0.22	Freeman3_odd	−0.10
Holm2_T3	0.38 **	Yamaguchi3_dbl	−0.22	Bar2_T2	−0.07
H_A_Alpha_T3	0.37 **	Huynen_T2	0.20	VanZyl3_odd	−0.02
Holm1_T3	0.37 **	Yamaguchi3_vol	−0.19	Yamaguchi4_hlx	0.00

Note: ** is significant at the 0.01 level and * is significant at the 0.05 level; _ground, _odd, and _T1 are surface scattering, _vol and _T2 are volume scattering, _dbl and _T3 are double-bounce scattering, and _hlx is helix scattering.

## Data Availability

Data will be made available on request; further inquiries can be directed to the corresponding author.
